# Health Benefits of Daily Walking on Mortality Among Younger-Elderly Men With or Without Major Critical Diseases in the New Integrated Suburban Seniority Investigation Project: A Prospective Cohort Study

**DOI:** 10.2188/jea.JE20140190

**Published:** 2015-11-05

**Authors:** Wenjing Zhao, Shigekazu Ukawa, Takashi Kawamura, Kenji Wakai, Masahiko Ando, Kazuyo Tsushita, Akiko Tamakoshi

**Affiliations:** 1 Department of Public Health, Hokkaido University Graduate School of Medicine, Sapporo, Japan; 1 北海道大学大学院医学研究科社会医学講座公衆衛生学分野; 2 Kyoto University Health Service, Kyoto, Japan; 2 京都大学保健管理センター; 3 Department of Preventive Medicine, Nagoya University Graduate School of Medicine, Nagoya, Japan; 3 名古屋大学大学院医学系研究科予防医学; 4 Center for Advanced Medicine and Clinical Research, Nagoya University Hospital, Nagoya, Japan; 4 名古屋大学附属病院先端医療臨床研究支援センター; 5 Comprehensive Health Science Center, Aichi Health Promotion Public Interest Foundation, Chita, Aichi, Japan; 5 あいち健康の森健康科学総合センター

**Keywords:** walking, mortality, younger elderly, secondary prevention

## Abstract

**Background:**

Regular physical activity contributes to the prevention of cancer, cardiovascular disease, and other chronic diseases. However, the frequency of physical activity often declines with age, particularly among the elderly. Thus, we investigated the effects of daily walking on mortality among younger-elderly men (65–74 years) with or without major critical diseases (heart disease, cerebrovascular disease, or cancer).

**Methods:**

We assessed 1239 community-dwelling men aged 64/65 years from the New Integrated Suburban Seniority Investigation Project. We estimated hazard ratios (HRs) of all-cause mortality and 95% confidence intervals (CIs) according to daily walking duration and adjusted for potential confounders, including survey year, marital status, work status, education, smoking and drinking status, BMI, regular exercise, regular sports, sleeping time, medical status, disease history, and functional capacity.

**Results:**

For men without critical diseases, mortality risk declined linearly with increased walking time after adjustment for confounders (*P*_trend_ = 0.018). Walking ≥2 hours/day was significantly associated with lower all-cause mortality (HR 0.49; 95% CI, 0.27–0.90). For men with critical diseases, walking 1–2 hours/day showed a protective effect on mortality compared with walking <0.5 hours/day after adjustment for confounders (HR 0.29; 95% CI, 0.06–1.20). Walking ≥2 hours/day showed no benefit on mortality in men with critical diseases, even after adjustment for confounders.

**Conclusions:**

Different duration of daily walking was associated with decreased mortality for younger-elderly men with or without critical diseases, independent of sociodemographic and lifestyle factors, BMI, medical status, disease history, and functional capacity. Incorporating regular walking into daily lives of younger-elderly men may improve longevity and successful aging.

## 要旨

**背景:**

定期的な運動は、がん、心血管疾患および他の慢性疾患の予防に有用である。しかし、加齢に伴い運動習慣は減少し、特にその減少は高齢者で顕著である。そこで、本研究は、前期高齢者(65-74歳)を対象として重大な疾患（心疾患、脳血管疾患、またはがん）の既往歴有無別に、日常的な歩行習慣と総死亡の関連を検討することを目的とした。

**方法:**

**NISSIN Project(the New Integrated Suburban Seniority Investigation Project)に登録された**64または65歳の男性1239人を対象とし、調査年、配偶者の有無、就業状況、教育歴、喫煙習慣、飲酒習慣、BMI、定期的な運動の有無、定期的なスポーツの有無、睡眠時間、医学的状態、既往歴、および機能的能力を交絡因子として調整した日常的な歩行時間による総死亡のハザ－ド比(HRs)、および95%信頼区間(CIs)を推定した。

**結果:**

重大な疾患の既往歴がない男性では、交絡因子調整後も日常的な歩行時間の増加に伴って死亡リスクが直線的に減少した(P trend=0.018)。１日2時間以上歩行する者は、１日の歩行時間が30分以内の者と比較して全死亡リスクが有意に低かった(HR: 0.49, 95%CI: 0.27-0.90)。重大な疾患の既往歴がある者では、１日歩行時間が30分以内の者と比較して、1-2時間歩行する者で死亡リスクの減少傾向を認めた(HR: 0.29, 95% CI: 0.06-1.20)。一方、1日2時間以上の歩行者では、この関連は認めなかった。

**結論:**

前期高齢男性において、重大な疾患の既往歴の有無にかかわらず、日常的な歩行習慣は死亡リスク減少と関連があった。この関連は、社会人口学的要因やライフスタイル、BMI、医学的状態、既往歴、および機能的能力を含む交絡因子と独立していた。前期高齢男性において、定期的な歩行習慣を日常生活に組み込む事で、寿命を延長し、豊かな老いを迎えられる可能性が示唆された。

## INTRODUCTION

There is mounting evidence that regular physical activity is a good indicator of health-related outcomes later in life.^1^ Regular physical activity has been reported to positively contribute to primary and secondary prevention of cardiovascular disease, cancer, hypertension, diabetes, and other chronic disease events.^2^ However, age-related declines in physical activity are common,^3^ especially for elderly people with heart disease.^2^

Walking, a preferred moderate physical activity for the elderly because of its relative ease and accessibility,^4^ has been encouraged to be incorporated into the daily routines of aging populations to help maintain a physically active lifestyle.^5^^,^^6^ Walking has even been recommended for patients with existing diseases who are less likely to practice moderate-to-vigorous physical activity.^7^^,^^8^ Several reviews and meta-analyses have elucidated the positive associations between walking and cardiovascular disease events among the general population,^9^^–^^11^ and the effects of these benefits on all-cause or cardiovascular mortality in elderly populations have also been demonstrated.^12^^–^^14^ Collectively, looking at a broad spectrum of ages might fail to clarify the beneficial effects of walking on health-related issues among the elderly; it is unreasonable to apply the results observed among younger or middle-aged adults to an older population,^15^ as pre-existing illnesses in the study population might enhance the bias in terms of health outcomes.^9^ Moreover, although some previous studies have suggested that cardiovascular disease or diabetes patients are likely to obtain health benefits through regular walking,^11^ data on secondary prevention is still lacking. Examination of these issues can improve our understanding of the role walking may play in the presence or absence of established chronic conditions in an elderly population.

Therefore, the current study was designed to investigate the health benefits of daily walking on mortality among younger-elderly men (aged 65–74 years) with or without heart disease, cerebrovascular disease, or cancer to elucidate how walking could aid in successful aging and longevity.

## METHODS

### Study population

The data were derived from the New Integrated Suburban Seniority Investigation Project, an on-going age-specific prospective cohort study. The rationale and design of the study has been described previously in detail.^16^ Briefly, based on the basic resident registry, community-dwelling elderly who were age 64 at the beginning of each given survey year in a city in central Japan were invited via letter to take part in a free comprehensive medical examination and complete a self-administrated questionnaire each June from 1996 through 2005. A total of 3073 participants (1548 men and 1525 women) agreed to register as cohort members. In the present study, 1239 men were selected as the study sample, after excluding those who entered the study after 2003 (304 subjects) because their 10-year follow-up had not been completed, and men with missing data on daily walking (5 subjects).

Consent to complete the questionnaire was obtained orally by an opt-out approach from 1996 to 2001, and in writing by an opt-in approach thereafter.^16^ The study protocol was approved by the Ethics Committees of Nagoya University Graduate School of Medicine, the National Center for Geriatrics and Gerontology of Japan, Aichi Medical University School of Medicine, and Hokkaido University Graduate School of Medicine.

### Baseline assessment

Baseline data were obtained through self-administered questionnaires and objective measurements taken during the health examination. Walking duration was assessed based on self-reported time spent daily walking by asking “How many hours a day do you walk?”. The available options included <30 minutes/day, 30 minutes to 1 hour/day, 1–2 hours/day and ≥2 hours/day. Time spent on daily walking was clearly defined in the questionnaire to include walking for exercise, working, and household, social, or other activities. The term “major critical diseases” was defined as any heart disease, cerebrovascular disease, or cancer (the three leading causes of death in Japan), which account for more than 50% of the mortality of elderly aged 65 years.^17^ Information on these diseases was also obtained from the questionnaire, in which subjects recorded their current treatment situation for existing diseases and the age when a physician diagnosed them with these diseases.

Sociodemographic and lifestyle factors included marital status (married, divorced, widow, single, or other), education (elementary school, junior high school, high school, junior college, university, or other), work status (not working or working), smoking status (never, past, or current), drinking status (never or current), regular exercise (never, <once/week, or ≥once/week), participation in regular sports (yes or no), and sleeping time (<7, 7–8, or >8 hours/day). Body mass index (BMI) was calculated and categorized into three groups (<18.5, 18.5–24.9, or ≥25 kg/m^2^). Medical status included hypertension, hyperlipidaemia, and diabetes mellitus. Hypertension was defined as a measured systolic blood pressure ≥140 mm Hg, diastolic blood pressure ≥90 mm Hg, self-reported hypertension, or use of antihypertensive medication. Hyperlipidaemia was defined as a total cholesterol level ≥220 mg/dL or self-reported hyperlipidaemia. Diabetes mellitus was defined as haemoglobin A1c (HbA1c) ≥6.5% (according to the National Glycohemoglobin Standardization Program [NGSP] method), fasting plasma glucose ≥126 mg/dL, or self-reported diabetes mellitus. In the New Integrated Suburban Seniority Investigation Project, HbA1c was measured according to Japan Diabetes Society (JDS) units. We transformed these results to NGSP values based on the conversion formula: NGSP (%) = 1.02 * JDS (%) + 0.25%.^18^ History of disease included self-reported history of clinically recognized chronic bronchitis, neuralgia/osphyalgia, or arthritis, or use of medication for these diseases. The short-form Geriatric Depression Scale (GDS-15) is a self-rated depression-screening tool for the elderly, and a total score of 6 or higher was treated as significant depressive tendency.^19^^–^^21^ The Tokyo Metropolitan Institute of Gerontology (TMIG) Index of Competence was used to evaluate intellectual, physical, and social function capacity, and possible total scores ranged from 0 to 13, with a higher score indicating a greater competence level.^22^ Gait speed was used to assess physical function as in previous studies, where it has been applied to evaluate the physical function of elderly populations with intellectual disability^23^ and community-dwelling populations.^24^ Gait speed was categorized into three groups (slow, normal, and fast) by asking, “How fast have you walked over the past year?”. Although our data were self-reported gait speed rather than objective gait speed, we checked the validity of self-reported gait speed in our previous study.^25^

### Follow-up

Dates for death and relocation out of the city were obtained from the basic resident registry. All participants were followed until death from any cause, relocation, or the last day of the year when they reached 75 years of age, whichever occurred first.

### Statistical analysis

Descriptive statistics were used to summarize the baseline characteristics of participants according to time spent daily walking and the morbidity of major critical diseases (Table 1). If the data were continuous, they were categorized according to standard clinical reference values. A Cox proportional hazard model was applied to estimate the hazard ratios (HRs) and 95% confidence intervals (CIs) for all-cause mortality based on time spent walking for men with or without major critical diseases. The proportional hazard assumption was tested by log-log plots. The interaction between daily walking and exercise, or daily walking and sports participation, was examined by the likelihood-ratio test. In multivariate analysis, we created three models to adjust for potential confounders. Model I adjusted for survey year; model II included survey year, sociodemographic and lifestyle factors (marital status, working status, education, smoking and drinking status, participation in regular exercise, regular sports, and sleeping time), and BMI; and model III adjusted for all the confounders in model II as well as medical status (hypertension, hyperlipidaemia, and diabetes mellitus), history of disease (chronic bronchitis, neuralgia/osphyalgia, and/or arthritis), and functional capacity (gait speed, GDS-15, and TMIG scores). Sensitivity analyses were conducted by excluding subjects who died or that dropped out of the study for any reason (eg, relocated or refused follow-up) within the first three years (model IV), or by excluding subjects who were diagnosed with heart disease, cerebrovascular disease, or cancer at age 63, 64, or 65 (model V). We also tested for linear trends in the categories of time spent walking in the Cox proportional hazard models.

**Table 1.** Baseline characteristics according to daily walking and morbidity of major critical diseases (*n* = 1239)

	Without major critical diseases (*n* = 986)				With major critical diseases (*n* = 253)			
	
	<0.5 h/d (*n* = 182)	0.5–1 h/d (*n* = 338)	1–2 h/d (*n* = 245)	≥2 h/d (*n* = 221)	<0.5 h/d (*n* = 56)	0.5–1 h/d (*n* = 100)	1–2 h/d (*n* = 54)	≥2 h/d (*n* = 43)
Marital status								
Married	178 (97.8)	321 (95.0)	228 (93.1)	204 (92.3)	54 (96.4)	98 (98.0)	54 (100.0)	42 (97.7)
Other	3 (1.6)	17 (5.0)	17 (6.9)	17 (7.7)	2 (3.6)	2 (2.0)	—	1 (2.3)
Work status								
Not working	87 (47.8)	135 (39.9)	116 (47.3)	64 (29.0)	32 (57.1)	46 (46.0)	28 (51.9)	14 (32.6)
Working	94 (51.6)	201 (59.5)	128 (52.2)	153 (69.2)	23 (41.1)	51 (51.0)	26 (48.1)	28 (65.1)
Highest education								
Junior school or less	46 (25.3)	87 (25.7)	69 (28.2)	103 (46.6)	13 (23.2)	17 (17.0)	15 (27.8)	15 (34.9)
High school	76 (41.8)	127 (37.6)	84 (34.3)	81 (36.7)	27 (48.2)	41 (41.0)	16 (29.6)	18 (41.9)
College or more	60 (33.0)	124 (36.7)	91 (37.1)	37 (16.7)	16 (28.6)	42 (42.0)	23 (42.6)	10 (23.3)
Smoking status								
Never	35 (19.2)	64 (18.9)	47 (19.2)	48 (21.7)	10 (17.9)	20 (20.0)	8 (14.8)	6 (14.0)
Past	86 (47.3)	170 (50.3)	104 (42.4)	85 (38.5)	28 (50.0)	56 (56.0)	31 (57.4)	25 (58.1)
Current	61 (33.5)	104 (30.8)	93 (38.0)	88 (39.8)	18 (32.1)	24 (24.0)	15 (27.8)	12 (27.9)
Drinking status								
Never	58 (31.9)	109 (32.2)	71 (29.0)	65 (29.4)	20 (35.7)	31 (31.0)	19 (35.2)	17 (39.5)
Current	124 (68.1)	229 (67.8)	174 (71.0)	156 (70.6)	36 (64.3)	69 (69.0)	35 (64.8)	25 (58.1)
BMI (kg/m^2^)								
<18.5	13 (7.1)	9 (2.7)	13 (5.3)	10 (4.5)	1 (1.8)	6 (6.0)	2 (3.7)	1 (2.3)
18.5–25	121 (66.5)	255 (75.4)	184 (75.1)	162 (73.3)	35 (62.5)	68 (68.0)	40 (74.1)	29 (67.4)
≥25	48 (26.4)	74 (21.9)	48 (19.6)	49 (22.2)	20 (35.7)	26 (26.0)	12 (22.2)	13 (30.2)
Regular exercise								
Never	99 (54.4)	115 (34.2)	78 (31.8)	109 (49.3)	35 (62.5)	32 (32.0)	13 (24.1)	19 (44.2)
<once/week	25 (13.7)	37 (11.0)	22 (9.0)	21 (9.5)	5 (8.9)	6 (6.0)	2 (3.7)	5 (11.6)
≥once/week	58 (31.9)	184 (54.8)	145 (59.2)	91 (41.2)	16 (28.6)	62 (62.0)	39 (72.2)	19 (44.2)
Regular sports								
No	92 (50.8)	141 (42.3)	98 (40.0)	129 (58.4)	39 (70.9)	42 (42.0)	19 (35.2)	21 (48.8)
Yes	89 (49.2)	192 (57.7)	144 (58.8)	90 (40.7)	16 (29.1)	58 (58.0)	35 (64.8)	22 (51.2)
Sleeping time								
<7 h/d	86 (47.3)	157 (46.6)	101 (41.2)	101 (45.7)	23 (41.8)	41 (41.0)	19 (35.2)	15 (34.9)
7–8 h/d	70 (38.5)	139 (41.2)	113 (46.1)	89 (40.3)	21 (38.2)	47 (47.0)	29 (53.7)	23 (53.5)
≥8 h/d	26 (14.3)	41 (12.2)	31 (12.7)	31 (14.0)	11 (20.0)	12 (12.0)	6 (11.1)	5 (11.6)
Medical status								
Hypertension	100 (54.9)	170 (50.3)	134 (54.7)	110 (49.8)	28 (50.0)	57 (57.0)	30 (55.6)	23 (53.5)
Hyperlipidemia	66 (36.3)	116 (34.4)	91 (37.1)	68 (30.8)	15 (27.3)	31 (31.0)	19 (35.2)	14 (33.3)
Diabetes Mellitus	27 (14.9)	45 (13.4)	50 (20.4)	21 (9.5)	12 (21.8)	21 (21.0)	12 (22.2)	5 (11.9)
History of disease	26 (14.3)	43 (12.7)	28 (11.4)	25 (11.3)	8 (14.3)	15 (15.0)	3 (5.6)	5 (11.6)
Gait speed								
Slow	44 (24.3)	33 (9.8)	16 (6.5)	20 (9.1)	16 (29.1)	13 (13.0)	10 (18.5)	3 (7.0)
Normal	125 (69.1)	250 (74.0)	193 (78.8)	168 (76.0)	38 (69.1)	77 (77.0)	38 (70.4)	34 (79.1)
Fast	12 (6.6)	55 (16.3)	36 (14.7)	33 (14.9)	1 (1.8)	10 (10.0)	6 (11.1)	6 (14.0)
GDS-15 score								
<6	139 (76.4)	276 (81.7)	197 (80.4)	182 (82.4)	36 (65.5)	77 (77.0)	46 (85.2)	34 (79.1)
≥6	43 (23.6)	62 (18.3)	47 (19.2)	35 (15.8)	19 (34.5)	23 (23.0)	8 (14.8)	9 (20.9)
TMIG score								
≤10	33 (18.1)	42 (12.4)	34 (13.9)	27 (12.2)	10 (18.2)	13 (13.0)	6 (11.1)	9 (20.9)
>10	149 (81.9)	296 (87.6)	211 (86.1)	193 (87.3)	45 (81.8)	87 (87.0)	48 (88.9)	34 (79.1)

GDS-15, Geriatric Depression Scale (Short Form); h/d, hours/day; TMIG, Tokyo Metropolitan Institute of Gerontology Index of Competence.

All values shown are as *n* (%). The proportion of each variable does not always add up to 100% owing to missing data.

All *P* values were based on two-tailed tests of significance; *P* < 0.05 was taken to be statistically significant. All statistical analyses were performed using JMP Clinical 5 for Microsoft Windows (SAS Institute Inc., Cary, NC, USA).

## RESULTS

During the total 12 829 person-years of follow-up, 175 men died. Table 1 summarizes the baseline characteristics of the participants according to daily time spent walking and absence or presence of major critical diseases. Of all 1239 men, 253 reported a history of heart disease (161 events), cerebrovascular disease (67 events), or cancer (42 events). For men without major critical diseases, those walking ≥2 hours/day were less likely to be married and more likely to have a job, a lower education level, and a lower prevalence of diabetes mellitus, and walked faster than men walking <0.5 hours/day. For men with major critical diseases, those who walked 1–2 hours/day had a higher education level, performed more regular exercise and sports per week, had a lower prevalence of diabetes mellitus, and had fewer depressive symptoms and higher TMIG scores than men in other walking groups. Table 2 presents the HRs of daily walking on mortality and 95% CIs for all the males. After adjustment for survey year, sociodemographic and lifestyle factors, BMI, medical status, history of disease, and functional capacity, walking ≥2 hours/day was significantly associated with all-cause mortality in all men (HR 0.52; 95% CI, 0.30–0.88), and the association displayed a linear trend (*P*_trend_ = 0.009).

**Table 2.** Association of daily walking with mortality in all participants

	<0.5 h/d	0.5–1 h/d	1–2 h/d	≥2 h/d	*P* for trend
Person-years	2379	4558	3100	2792	
Deaths	42	67	40	26	
Model I	1.00	0.82 (0.56, 1.22)	0.73 (0.47, 1.12)	0.53 (0.32, 0.86)*	0.010*
Model II	1.00	0.87 (0.59, 1.32)	0.72 (0.45, 1.13)	0.49 (0.29, 0.82)*	0.005*
Model III	1.00	0.95 (0.63, 1.46)	0.79 (0.49, 1.27)	0.52 (0.30, 0.88)*	0.009*

CI, confidence interval; h/d, hours/day; HR, hazard ratio.

Values shown are HR (95% CI). *P < 0.05.

Model I adjusted for survey year. Model II adjusted for survey year, marital status, work status, education, smoking and drinking status, BMI, regular exercise, daily sleeping duration, and regular sports. Model III adjusted for the confounders in model II as well as hypertension, hyperlipidaemia, diabetes mellitus, chronic bronchitis, neuralagia/osphyalgia, arthritis, gait speed, and GDS-15, and TMIG scores.

Table 3 shows the HRs of daily walking on mortality and 95% CIs stratified by those with or without major critical diseases. For men without major critical diseases, the risk of death linearly declined as walking duration increased after adjustment for survey year, sociodemographic and lifestyle factors, and BMI (*P*_trend_ = 0.012). Further adjustment for medical status, history of disease, and functional capacity did not alter the initial trend (*P*_trend_ = 0.018) and led to a significant association of mortality with walking ≥2 hours/day (HR 0.49; 95% CI, 0.27–0.90). Men with major critical diseases presented a different association from the total sample of men. Walking 1–2 hours/day showed a protective effect on mortality after adjustment for survey year, sociodemographic and lifestyle factors, and BMI (model II: HR 0.30; 95% CI, 0.07–1.11) compared with walking <0.5 hours/day. Following adjustment for medical status, history of disease, and functional capacity (model III), walking 1–2 hours/day was still associated with decreased mortality (HR 0.29; 95% CI, 0.06–1.20). Walking ≥2 hours/day did not show substantial benefits for mortality in men with major critical diseases after full adjustment for confounding factors (HR 0.82; 95% CI, 0.20–3.24), and a linear trend was not observed.

**Table 3.** Association of daily walking with mortality according to morbidity of major critical diseases

	Without major critical diseases				*P* for trend	With major critical diseases				*P* for trend
	
	<0.5 h/d	0.5–1 h/d	1–2 h/d	≥2 h/d		<0.5 h/d	0.5–1 h/d	1–2 h/d	≥2 h/d	
Person-years	1821	3534	2509	2330		558	1024	592	462	
Death Case	32	51	36	20		10	16	4	6	
Model I	1.00	0.82 (0.53, 1.28)	0.83 (0.52, 1.35)	0.50 (0.28, 0.86)*	0.023*	1.00	0.85 (0.38, 2.04)	0.35 (0.09, 1.07)	0.75 (0.25, 2.10)	0.189
Model II	1.00	0.94 (0.59, 1.50)	0.85 (0.51, 1.41)	0.48 (0.26, 0.86)*	0.012*	1.00	0.98 (0.37, 2.76)	0.30 (0.07, 1.11)	0.69 (0.19, 2.38)	0.129
Model III	1.00	1.03 (0.64, 1.68)	0.94 (0.56, 1.60)	0.49 (0.27, 0.90)*	0.018*	1.00	0.92 (0.31, 2.94)	0.29 (0.06, 1.20)	0.82 (0.20, 3.24)	0.289

Person-years	1806	3504	2485	2312		555	1016	588	460	
Death Case	28	45	31	15		10	14	3	5	
Model IV	1.00	1.01 (0.61, 1.70)	0.92 (0.53, 1.63)	0.40 (0.20, 0.76)*	0.006*	1.00	0.78 (0.26, 2.53)	0.20 (0.03, 0.93)*	0.48 (0.11, 2.02)	0.078

Person-years	—	—	—	—	—	446	705	452	367	
Death Case	—	—	—	—	—	8	9	3	4	
Model V^a^	—	—	—	—	—	1.00	0.73 (0.21, 2.54)	0.17 (0.03, 0.96)*	0.65 (0.11, 3.20)	0.289

CI, confidence interval; h/d, hours/day; HR, hazard ratio.

Values shown are HR (95% CI); *P < 0.05.

^a^HR and 95% CI are not shown for men without diseases because these values were equal to those in model III.

Model I adjusted for survey year. Model II adjusted for survey year, marital status, work status, education, smoking and drinking status, BMI, regular exercise, daily sleeping duration, and regular sports. Model III adjusted for the confounders in model II as well as hypertension, hyperlipidaemia, diabetes mellitus, chronic bronchitis, neuralagia/osphyalgia, arthritis, gait speed, and GDS-15 and TMIG scores. Model IV excluded subjects who died or were censored within the first three years, and adjusted for all the confounders in model III. Model V excluded subjects who were diagnosed with heart disease, cerebrovascular disease, or cancer at the age of 63, 64, or 65 years and adjusted for all the confounders in model III.

In the sensitivity analyses, after excluding participants who died or that were censored within the first 3 years (model IV), walking ≥2 hours/day was still inversely associated with mortality among men without major critical diseases (HR 0.40; 95% CI, 0.20–0.76), and significantly decreased mortality was observed among those with major critical diseases within the intermediate walking duration of 1–2 hours/day (HR 0.20; 95% CI, 0.03–0.93). After excluding subjects who suffered from major critical diseases at the age of 63, 64, or 65 (model V), this association did not change substantially among men with major critical diseases (HR 0.17; 95% CI, 0.03–0.96). In addition, we did not observe any significant interactions on mortality between daily walking and exercise (*P* = 0.318) or participation in sports (*P* = 0.229).

## DISCUSSION

After follow-up to the age of 75, we observed that daily walking was associated with a decreased risk of death for younger-elderly Japanese men, independent of sociodemographic and lifestyle factors, BMI, medical status, history of disease, and functional capacity. There was a linear association between daily walking and all-cause mortality among men without major critical diseases, while there was a peak in benefit at an intermediate duration of daily walking for men with major critical diseases.

To our knowledge, this is the first age-specific cohort study to elucidate the effect of daily walking on mortality among younger-elderly men, stratified by the presence and absence of heart disease, cerebrovascular disease, and cancer. Our findings are consistent with the few existing reports that mortality from cardiovascular disease,^26^^,^^27^ cancer,^28^ or all causes^28^^,^^29^ among older men was decreased with increasing walking distance or duration. Recent evidence suggests that daily walking could protect against all-cause mortality among elderly aged ≥65 years.^30^ Physically active participants have been reported to have low levels of inflammatory markers (serum high-sensitivity C-reactive protein and interleukin-6) that might be responsible for cardiovascular disease or other age-related diseases.^31^ It has been suggested that walking could contribute to improved cardiovascular fitness, reduced body weight and body fat, and decreased resting diastolic blood pressure, as well as influence lipid profiles, clotting factors, and other concomitant risk factors.^26^^,^^32^ Moreover, circulatory insulin-like growth factor I was reported to be inversely related to time spent walking among men, which may partly account for the protective effects of physical activity against cancer at certain sites in the body.^33^

The evidence is still unclear in terms of the benefits of time spent walking on mortality among the elderly with major critical diseases, although a minimum amount and type of physical activity has been proposed by the American College of Sports Medicine (ACSM) and the American Heart Association (AHA) for elderly individuals with chronic conditions to prevent other conditions from developing and assist with therapy for the documented diseases.^34^ Our findings provide new evidence that an intermediate walking duration (1–2 hours/day) can decrease mortality risk by 70% in younger-elderly community-dwelling men with cardiovascular disease, cerebrovascular disease, or cancer, while walking for a longer duration (≥2 hours/day) may not provide any significant benefits in these men. In previous studies in elderly populations, the largest category of daily walking was ≥1 hour/day, so the threshold of walking duration in the elderly with major critical diseases might not be clear.^7^^,^^10^^,^^29^ Our result is consistent with physical activity recommendations from the ACSM and AHA that older people with chronic diseases should tailor their activity plan according to the prescription from physicians to avoid the risk of inappropriate physical activity.^34^

There are also some studies concluding the same trend as our findings regarding the association between physical activity intensity and mortality, though there are no previous studies focusing on the association of walking duration with mortality for older populations with chronic diseases. For example, the British Regional Heart Study has demonstrated that regular light or moderate intensity physical activity, such as walking, is likely to be sufficient to achieve significant benefits, while sports or vigorous activities conferred no additional benefit for older men with established cardiovascular disease^35^ and coronary heart disease.^36^ A systematic review concerning cancer survivors showed that decreased mortality from colon cancer was associated with 6 hours of walking per week, and more vigorous exercise may not improve survival.^37^ However, the precise mechanism explaining why long duration of physical activity confers no additional benefits for the elderly is still not fully understood.^38^ Presumably, in the context of age- and disease-related heterogeneity in cardiorespiratory capacity, musculoskeletal fitness, medical comorbidities, and performance in activities of daily living, an intermediate walking duration, regardless of walking intensity, might be an optimal amount of time for men with major critical diseases. This amount of time might be appropriate for their capacity to undertake physical activities, while any longer is unlikely to yield any mortality benefit.

### Strengths and limitations

Our age-specific cohort study effectively eliminated the influence of age on mortality because age is a major risk factor for mortality and reduced physical activity.^1^ We conducted an extensive range of adjustments for sociodemographic and lifestyle factors, BMI, medical status, history of disease, and functional capacity, which isolated, as far as possible, an independent association between daily walking duration and mortality.^4^ We also checked the interaction between walking and exercise, as well as walking and regular sports, and adjusted for these two kinds of physical activity to keep the independent association between walking and all-cause mortality.

Some limitations have to be taken into account. First, as a prospective cohort study rather than a randomized clinical trial, this study is less likely to show true causality because the health status of the elderly participants might produce certain walking habits at baseline or change the level of daily walking over time, especially for participants with established major critical diseases who may have contraindications to participating in physical activities during the initial stage of disease diagnosis. However, to avoid reverse causality, we adopted two approaches to support this association. The first was to exclude participants who died or were censored within the first 3 years, thereby eliminating the bias caused by participants at higher risk of death. The second approach was to check the age at diagnosis for cardiovascular disease, cerebrovascular disease, and cancer, and then exclude those subjects diagnosed with one or more of these diseases at 63, 64, or 65 years of age, thereby decreasing the possibility that walking duration changed in those with major critical diseases. Second, the sample size for men with major critical diseases was small compared with previous analogous studies that involved physical activity in patients with established cardiovascular disease or cancer and covered a wide range of ages in the elderly. However, our study paid specific attention to a sub-domain of physical activity (regular walking) and restricted inclusion to the younger-elderly, which as a whole limited the enrolment of subjects. Meanwhile, due to the small sample size of this subgroup, the power of the test may be low, which may lead to a high possibility of type II error. Further study is needed to understand this association, with more attention focused on the younger-elderly with one or more chronic diseases. Third, our data on walking duration came from a self-administrated questionnaire rather than an objective measurement, and the validity of the single-item questionnaire was not assessed. Self-reported daily walking duration may be overestimated.^39^ Finally, information about walking intensity was lacking in our survey; however, this will be addressed in a future study.

### Conclusion

Our findings here suggest a theoretical walking duration for younger-elderly men with or without major critical diseases. Walking for relatively long periods was associated with a decrease in all-cause mortality for men without major critical diseases, while an intermediate walking duration was beneficial to younger-elderly men with major critical diseases. These findings support the view that daily walking has the potential to contribute to primary and secondary prevention of heart disease, cerebrovascular disease, cancer, and other diseases, and might be a promising and effective prescription of medicine for elderly with chronic conditions. This may also provide evidence for public health recommendations that incorporating regular walking into daily life may improve quality of life among the elderly and extend longevity. Younger-elderly men with one or more chronic conditions should gradually and regularly practice walking based on individual abilities and fitness to improve management of existing diseases and overall survival.

## ONLINE ONLY MATERIAL

Abstract in Japanese.
