# Supplementary material for: Health Benefits of Daily Walking on Mortality Among Younger-Elderly Men With or Without Major Critical Diseases in the New Integrated Suburban Seniority Investigation Project: A Prospective Cohort Study
Source: J Epidemiol. 2015 Nov 5;25(10):609–16. doi: 10.2188/jea.JE20140190 (PMC4626390; doi:10.2188/jea.JE20140190)
Supplement: Abstract in Japanese. [file je-25-609-s001.pdf]

前期高齢者における死亡に対する日常的な歩行習慣の健康効果—重大な疾患の既往歴有無別に—：NISSIN Project

趙文静[1]、 鵜川重和[1]、川村孝[2]、若井建志[3]、安藤昌彦[4]、津下一代[5]、玉腰暁子[1]

[1]北海道大学大学院医学研究科社会医学講座公衆衛生学分野
[2]京都大学保健管理センター
[3]名古屋大学大学院医学系研究科予防医学
[4]名古屋大学附属病院先端医療臨床研究支援センター
[5]あいち健康の森健康科学総合センター

要旨

背景　定期的な運動は、がん、心血管疾患および他の慢性疾患の予防に有用である。しかし、加齢に伴い運動習慣は減少し、特にその減少は高齢者で顕著である。そこで、本研究は、前期高齢者(65-74 歳)を対象として重大な疾患（心疾患、脳血管疾患、またはがん）の既往歴有無別に、日常的な歩行習慣と総死亡の関連を検討することを目的とした。

方法　NISSIN Project(the New Integrated Suburban Seniority Investigation Project)に登録された 64 または 65 歳の男性 1239 人を対象とし、調査年、配偶者の有無、就業状況、教育歴、喫煙習慣、飲酒習慣、BMI、定期的な運動の有無、定期的なスポーツの有無、睡眠時間、医学的状態、既往歴、および機能的能力を交絡因子として調整した日常的な歩行時間による総死亡のハザード比(HRs)、および 95%信頼区間(CIs)を推定した。

結果　重大な疾患の既往歴がない男性では、交絡因子調整後も日常的な歩行時間の増加に伴って死亡リスクが直線的に減少した(P trend=0.018)。１日２時間以上歩行する者は、１日の歩行時間が 30 分以内の者と比較して全死亡リスクが有意に低かった(HR: 0.49, 95%CI: 0.27-0.90)。重大な疾患の既往歴がある者では、１日歩行時間が 30 分以内の者と比較して、1-2 時間歩行する者で死亡リスクの減少傾向を認めた(HR: 0.29, 95% CI: 0.06-1.20)。一方、１日２時間以上の歩行者では、この関連は認めなかった。

結論　前期高齢男性において、重大な疾患の既往歴の有無にかかわらず、日常的な歩行習慣は死亡リスク減少と関連があった。この関連は、社会人口学的要因やライフスタイル、BMI、医学的状態、既往歴、および機能的能力を含む交絡因子と独立していた。前期高齢男性において、定期的な歩行習慣を日常生活に組み込む事で、寿命を延長し、豊かな老いを迎えられる可能性が示唆された。

キーワード: 歩行、死亡率、前期高齢者、二次予防
